# Bifurcation analysis of “synchronization fluctuation”: a diagnostic measure of brain epileptic states

**DOI:** 10.3389/fncom.2014.00011

**Published:** 2014-02-06

**Authors:** Fatemeh Bakouie, Keivan Moradi, Shahriar Gharibzadeh, Farzad Towhidkhah

**Affiliations:** ^1^Neural and Cognitive Sciences Lab, Biomedical Engineering Faculty, Amirkabir University of TechnologyTehran, Iran; ^2^Cybernetics and Modeling of Biological Systems Laboratory, Biomedical Engineering Faculty, Amirkabir University of TechnologyTehran, Iran

**Keywords:** complex network, synchronization fluctuation, dynamical system, bifurcation, control parameter

The brain is a complex network with functional elements spatially distributed in different regions. One suggested mechanism for communication among these distributed elements is synchronization (Singer, 1993).

Two oscillating neural groups are called to be “synchronized” if over the time, their phase difference does not remarkably increase. In a real system composed of some oscillators, synchronization level is a computable parameter. According to this paradigm, depending on the functional state of the brain, the level of synchronization among brain regions may vary over time. This variation is called “synchronization fluctuation” (SF). Regarding brain's higher functions such as consciousness and memory, for instance, SF patterns are important features of normal brain states (Schnitzler and Gross, 2005; Watrous et al., 2013).

In some pathological brain states such as epilepsy, however, hyper-synchronization is a major problem (Lehnertz et al., 2009). In such situations, synchronization occurs without fluctuations. Therefore, in epilepsy, SF may lose its dynamicity, producing a narrow-dynamics signal. The question which arises is: “how is it possible to manage diseases related to the poor dynamics of SF in the brain?”

Dynamical systems approach may be able to provide some answers to this question: Based on dynamical systems theory, even slight modification of a parameter (so-called “control parameter”) is able to lead to a significant qualitative change in the system's behavior. This change is called a “bifurcation” (Guckenheimer, 2007). Dynamical approach has already been successfully used to the study of the functional status of epileptic states. For example, Babloyantz and Destexhe reported the nonlinearity of absence (Babloyantz and Destexhe, 1986). Moreover, Stam claims that epilepsy is the most important application of nonlinear EEG study (Stam, 2005). In another research Perez Velazquez et al. suggested that the interictal ictal transition may be the result of bifurcation due to alteration in control parameters like the balance between excitation an inhibition in the underlying neuronal networks (Perez Velazquez et al., 2003).

We hypothesize that SF may be a representative parameter of brain dynamics, which have identifiable bifurcations according to specific brain states. According to such approach, SF dynamics is supposed to change from a rich state to a narrower one, when brain changes from normal conscious to abnormal unconscious epileptic conditions.

Biologically, different mechanisms have already been suggested as the underlying basis of brain synchronization. For instance, it has been shown that gap junctions, coupling of neurons via long-term synaptic plasticity, interneurons, and rhythm generators of the brain such as the medial septum-diagonal band of Broca (MSDBB) may play a role in the synchronization between two neurons or more neuronal networks (Buzsáki, 2002). Such biological mechanisms that control synchronization can be considered as control parameters of SF in brain dynamics. For example, among these parameters, variations may exist in the number and permeability of gap junctions, the synaptic strength between two neurons, the distribution, frequency and strength of the GABA inhibition by interneurons, and the distribution, frequency and strength of excitation and inhibition of the cholinergic and GABAergic neurons of the MSDBB. Moreover, Margineanu and Klitgaard have already demonstrated that levetiracetam (LEV) antagonizes neuronal (hyper) synchronization, in the CA3 area of rat brain slices which is prone to epilepsy (Georg Margineanu and Klitgaard, 2000). In another research, Clemens showed that Valproate decreases EEG synchronization in idiopathic generalized epilepsy (Clemens, 2008).

Concerning connectivity among brain regions, Kay et al. explained that in treatment-responsive epileptic patients, compared to healthy controls, default Mode Network (DMN) connectivity does not reduce significantly; however, in treatment-resistant epileptic patients, there exists connectivity reduction compared to control group (Kay et al., 2013). In another study, the researchers showed DMN alterations in mesial temporal lobe epilepsy. Furthermore, Liao et al. have showed that in mesial temporal lobe epilepsy (mTLE) patients with hippocampal sclerosis (HS), there are reductions in functional and structural connectivity between hippocampal structures and their adjacent regions (Liao et al., 2011). Compared to the controls, it was shown that there is significant reduction in functional and structural connectivity between the posterior cingulate cortex (PCC)/precuneus (PCUN) and bilateral mesial temporal lobes (mTLs). Resting functional magnetic resonance imaging studies showed that in drug-resistant temporal lobe epilepsy, functional connectivity between the hippocampus, anterior temporal, precentral cortices and the default mode and sensorimotor networks reduces Based on their findings it would be claimed that the reduction in functional connectivity within the DMN in mTLE may be the result of the connection density reduction, leading to degeneration of structural connectivity (Voets et al., 2012). These finding showed that in epilepsy, connectivity reduction occurred, while pharmacological treatment tend to drive this change in connectivity back to normal state. The mechanism of such therapeutic action, however, is still relatively unknown (Jin and Zhong, 2011).

In the future, it would be interesting to analyze the efficacy of therapeutic strategies addressing diseases caused by SF dynamicity changes (such as anti-epileptic drugs) according to their capacity to carefully tune the control parameters of SF in order to set the brain back to its normal states. As an evidence, Krystal et al. hypothesized that Lyapunov exponent (λ1) may decrease during the electroconvulsive therapy (ECT) seizures (Krystal et al., 1996). It seems that despite they did not assess synchronization directly, decreased λ1 corresponds to decreased EEG complexity. In another experimental treatment strategy for epilepsy, researchers have implemented an “automated, just-in-time stimulation seizure control method” in epileptic rats. Interestingly, the successful control of seizures with such therapy highly correlated with desynchronization of brain dynamics (Good et al., 2009).

Such experimental researches support the idea that, by tuning control parameters of SF, it may be possible to drive pathological brain states into normal ones. Therefore, we suggest that SF may be an important measure that represents the brain dynamics and that SF dynamics may be a potential subject of future experimental studies aiming to uncover the underlying mechanisms of pathological brain states.
